# Second primary cancers after radiation for prostate cancer: a review of data from planning studies

**DOI:** 10.1186/1748-717X-8-172

**Published:** 2013-07-08

**Authors:** Louise Murray, Ann Henry, Peter Hoskin, Frank-Andre Siebert, Jack Venselaar

**Affiliations:** 1 St James’s Institute of Oncology, Beckett St, Leeds LS9 7TF, UK; 2 University of Leeds, Leeds, UK; 3 Mount Vernon Cancer Centre, Northwood, London, UK; 4 Klinik fur Strahlentherapie, Kiel, Germany; 5 Institute Verbeeten, Tilburg, Netherlands

**Keywords:** Prostate cancer, Radiation induced second primary cancer, Radiotherapy techniques, Review

## Abstract

A review of planning studies was undertaken to evaluate estimated risks of radiation induced second primary cancers (RISPC) associated with different prostate radiotherapy techniques for localised prostate cancer. A total of 83 publications were identified which employed a variety of methods to estimate RISPC risk. Of these, the 16 planning studies which specifically addressed absolute or relative second cancer risk using dose–response models were selected for inclusion within this review. There are uncertainties and limitations related to all the different methods for estimating RISPC risk. Whether or not dose models include the effects of the primary radiation beam, as well as out-of-field regions, influences estimated risks. Regarding the impact of IMRT compared to 3D-CRT, at equivalent energies, several studies suggest an increase in risk related to increased leakage contributing to out-of-field RISPC risk, although in absolute terms this increase in risk may be very small. IMRT also results in increased low dose normal tissue irradiation, but the extent to which this has been estimated to contribute to RISPC risk is variable, and may also be very small. IMRT is often delivered using 6MV photons while conventional radiotherapy often requires higher energies to achieve adequate tissue penetration, and so comparisons between IMRT and older techniques should not be restricted to equivalent energies. Proton and brachytherapy planning studies suggest very low RISPC risks associated with these techniques. Until there is sufficient clinical evidence regarding RISPC risks associated with modern irradiation techniques, the data produced from planning studies is relevant when considering which patients to irradiate, and which technique to employ.

## Introduction and background

Prostate cancer (PCa) is the most common cancer in men in Europe and accounts for over one fifth of male cancer diagnoses [1]. Radiotherapy is one treatment option for localised and locally advanced PCa and may be delivered as external beam radiotherapy (EBRT), brachytherapy (BT) or combination EBRT and BT (EBRT-BT). Survival following radical radiotherapy has improved over the last decade, as a result of dose escalation and use of androgen deprivation. As survival improves, long term consequences of treatment become more relevant. One of the most serious long term effects following radiotherapy is development of a radiation induced second primary cancer (RISPC). Newer radiotherapy techniques such as IMRT have facilitated dose escalation, but differences in dose distribution and scatter have raised theoretical concerns about an increased risk of RISPC [2]. The potential risk of RISPC is particularly relevant in PCa: patients are now diagnosed at an earlier stage than in the past and so may receive treatment earlier, and patients are surviving for longer. As such, patients have a longer period in which RISPC may develop.

Some clinical data suggests that irradiated PCa patients may be at increased risk of RISPC, although the majority of clinical evidence concerns older EBRT techniques [3-12]. In terms of newer techniques, such as IMRT, BT and protons, clinical studies examining second primary cancers often have relatively low patient numbers and/or short durations of follow up [7,11,13-21]. Until further clinical information is available, planning studies provide theoretical RISPC risk estimates.

### Primary and secondary radiation

Radiation to normal tissues consists of primary radiation, the direct result of the treatment beams, as well as secondary radiation, which largely affects out-of-field tissues.

In photon treatments, secondary radiation results from scatter from within the patient and from the collimator, as well as leakage from the treatment machine [22-25]. Close to the target, scatter from within the patient is the main source of secondary radiation, while further from the target, leakage photons are important [22]. At higher photon energies (≥10MV), neutrons are produced from high density materials within the machine head and these may make a significant contribution to out-of field secondary dose [26].

For proton treatments, secondary radiation consists of secondary photons and neutrons produced in the patient and treatment head, and which indirectly contribute to out-of-field dose [27]. The relative biological effect, and thus appropriate radiation weighting factor, that should be applied to secondary neutrons is a matter of debate [28]. Secondary neutron production is influenced by proton delivery technique: spot scanned therapy uses magnets to direct the beam across a target, while passive scattering uses a scattering material to ‘spread out’ the beam. The presence of the scattering material within the beam causes additional secondary neutron production which contributes to whole body dose [27,29].

### Modelling second malignancy risk

In low dose out-of-field regions, radiation protection models are appropriate for estimating RISPC risk. A risk co-efficient, which reflects the likelihood of developing a second cancer in a specific organ, is applied to the equivalent dose received by that organ. The risk co-efficient is stated in per cent per Sievert and therefore, as dose increases, the risk of second malignancy increases in a linear fashion. The linear relationship is based on atomic bomb survivors and on the understanding that cells exposed to lower radiation doses are damaged, but not killed (or sterilised), by radiation, and so maintain the potential for malignant transformation [2]. Risk co-efficients may be adjusted for age and/or the population under consideration. A dose and dose-rate effectiveness factor (DDREF), which adjusts for low dose and low dose rate situations, (i.e. <100 mGy or <0.01Gymin^-1^) may also be applied when estimating out-of-field RISPC risk in these settings [2,30-35].

**Figure 1:**
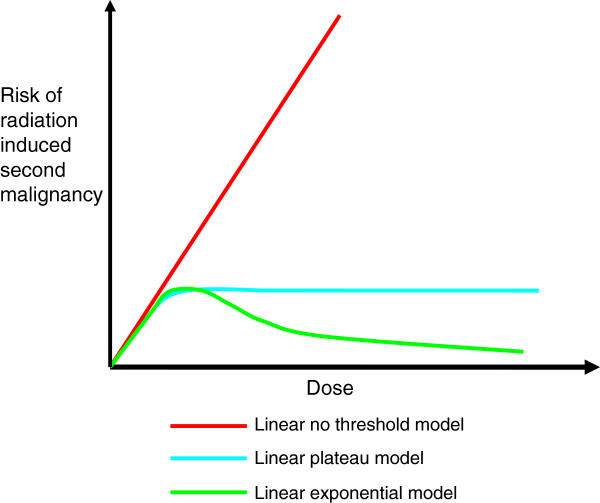
Illustration of traditional dose-risk models.

In higher dose regions the relationship between dose and risk of second cancer is less certain and a number of dose–response models are proposed. Models consider the balance between radiation induced cell damage, which leaves cells with the potential for malignant transformation, and cell sterilisation which renders cells incapable of transformation. The most commonly adopted models include the linear-no-threshold model (LNT), the linear-plateau (LP) model and the linear-exponential (LE) model. All three display a linear dose–response relationship for about the initial 4Gy of fractionated radiotherapy [2,36]. At higher doses there is variation: the LNT model presumes an on-going linear relationship at higher doses [37], the LP model presumes a plateau in risk beyond the linear portion of the curve, and the LE model suggests a reduction in the risk at higher doses as a result of increasing cell sterilisation. In reality, these models represent extremes, and it is likely that the true relationship lies somewhere between the LP and LE models [2]. Neither the LNT, LP nor LE models account for the effects of fractionation. These three models are illustrated in Figure 1.

A further model is the competitive risk model which also encompasses the effects of mutation induction at lower doses, and cell killing at higher doses. The relationship is approximately linear at lower doses, but then begins to fall at higher doses, according to a linear-quadratic function [38]. This model also accommodates inhomogeneous dose distributions within an organ and fractionation.

### Organ equivalent dose

The concept of organ equivalent dose (OED) states that any dose distribution in an organ that results in the same RISPC incidence as an alternative dose distribution, has the same OED. This concept has employed LE, LP and LNT models. Estimations of RISPC incorporate age at irradiation, attained age, gender, and fractionation. The model considers radiation from the primary beam and out-of field components, which is considered a positive and more realistic move away from models which only consider out-of-field doses [39,40]. OED is proportional to RISPC risk. The OED model is, however, based on a very specific cancer population (Hodgkin’s disease patients treated with radiotherapy and chemotherapy), and so the applicability of this model to other cancer populations has been questioned [39].

## Review

Planning studies were identified from literature review of Medline (from 1946), EMBASE (from 1947) and CENTRAL (from 1974) databases. Search terms related to RISPC, radiotherapy and PCa. The precise search terms from the Medline search are provided as Additional file 1. The last search was performed on January 16th 2012. References and “related articles” were also reviewed. 565 different articles were identified including 83 radiotherapy planning studies. Within these, various methods were employed to estimate RISPC risk. This review focuses on the 16 planning studies (and one more recent study) which specifically addressed absolute or relative RISPC risk using dose–response models. The protocol for this literature review was reviewed by the St James's Institute of Oncology Radiotherapy Research and Development Group but no formal ethical committee review was required as this was a review.

### Planning studies

Of the 16 studies, five evaluated out-of-field risk alone, without consideration of the impact of the primary beam. This approach has been criticised [37,41] as by neglecting the impact of the cell sterilisation component of the primary dose, it may overestimate RISPC. Equally, however, ignoring the effect of the primary dose could falsely decrease estimated RISPC risk if risk were actually to continue to increase beyond 4Gy, rather than plateau or decrease. Thus the impact of neglecting primary radiation dose is dependent of the risk model employed. There is considerable variation amongst the absolute risks reported from one study to the next. Differences in risk co-efficients, correction factors, the region studied (i.e. out-of-field alone or not) and dose–response models employed undoubtedly contribute. As the correct dose–response relationship is unknown, it is impossible to say which study has provided the most accurate estimates. Nonetheless, data within each study is valuable when comparing treatments. Many planning studies have included several dose-risk models in an effort to demonstrate the range of possible outcomes. To add to the uncertainty, methods of data collection have varied amongst studies. For example, when assessing out-of-field dose, some studies have measured doses using phantoms, some have used previously published measurements, while others have used Monte Carlo simulations. In terms of assessing dose from the primary beam, a variety of planning systems and algorithms have been employed. These factors may also contribute to the heterogeneity in RISPC estimates.

### Impact of IMRT vs. 3D-CRT or conventional RT

**Figure 2:**
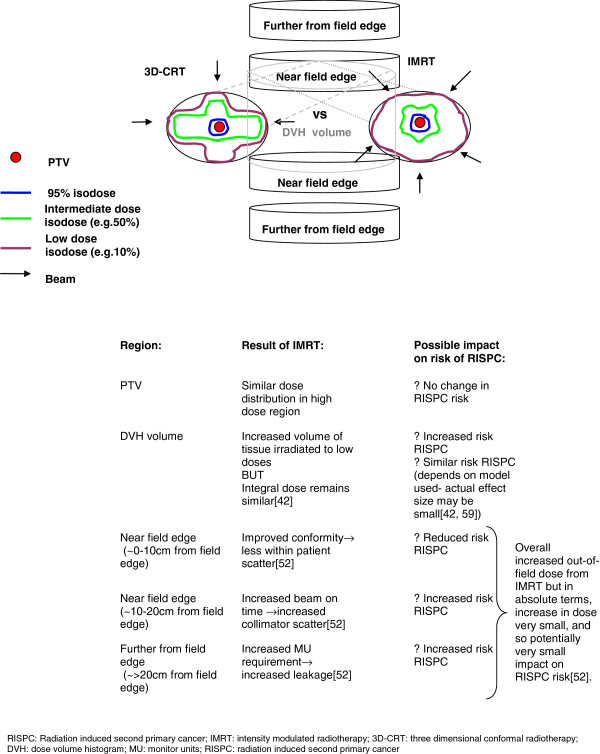
Illustration of factors which may impact on the risk of radiation induced second primary cancers when using IMRT instead of 3D-conformal radiotherapy.

Factors of importance when considering the impact of IMRT on RISPC risk compared to 3D-CRT or conventional radiotherapy include the consequence of a change in dose distribution and the increase in monitor units (MU) required to deliver treatment (Figure 2). The different dose distribution has two potential effects. Firstly, a larger volume of normal tissue is irradiated to lower doses, which may contribute to increased RISPC risk in in-field tissues and in tissues in the immediate vicinity (i.e. tissues within the DVH volume, that is those included with the CT planning scan volume; [2,37,42]). The impact of this on RISPC risk is influenced by the dose model employed: in theory the LE model predicts increased RISPC risk as a result of the low dose spread from IMRT, compared to the relatively high doses and lack of low dose spread delivered with 3D-CRT (i.e. the majority of normal tissue dose from 3D-CRT is likely to fall further along the downward part of the LE curve). According to the LNT model, which predicts that RISPC risk will increase with dose, tissues receiving low dose spread from IMRT which would otherwise not have received such a dose with 3D-CRT, will have a higher RISPC risk. With an LP model, the impact of low dose spread will depend on whether that dose falls on the linear part of the curve (where risk increases with increasing dose) or the plateau part (where risk remains stable). Secondly, however, the improved conformity of IMRT, and frequently accompanying smaller field sizes, may result in reduced scatter in nearby out-of-field tissues (i.e. tissues 15-30 cm from the field edge), thus reducing RISPC risk [42-44]. IMRT delivery requires increased MU resulting in increased machine leakage leading to increased out-of-field dose in tissues further from the field, which also contributes to RISPC risk. The relative contribution of the above components determines the magnitude of RISPC risk. In terms of the high doses within the PTV, the risk of RISPC (in particular sarcoma) is thought to remain relatively unchanged when moving from 3D-CRT to IMRT as there is little change in the dose distribution within the target region itself [2].

With two exceptions [33,42], the reviewed studies suggest that IMRT results in increased RISPC risk (Table 1). In general, the magnitude of absolute risk has been estimated to be small, but can be more than double that estimated for conventional treatments, depending on the model and whether the primary beam is considered in addition to out-of-field dose [45,46].

**Table 1:** Risk of second fatal malignancy with IMRT compared to conventional or 3D-CRT

**Study**	**Region assessed**^**§**^	**Method of obtaining dose data**	**Method of risk calculation**	**Energy**	**% Risk of second fatal malignancy**	
					**Conventional/ 3D-CRT**	**IMRT**
Followill 1997 [47]^¥^	Out of field	Measured in phantom- previously published data	Whole body dose equivalent for neutrons and photons	6MV	0.6%§	1%
				18MV	2.5%	4.5%
			NCRP risk coefficients	25MV	4.5%	8.4%
Hall 2003 [2]	In field and out of field	Scanned volume: calculated from DVHs (TPS not stated)	LP	6MV	1%	1.75%
			LE (with 2 gradients of dose fall off)			
		Scatter: measured in phantom- previously published				
Kry 2005 [40]	Out of field	Measured in phantom- previously published data	Organ specific dose equivalents for photons and neutrons	6MV	NR	2.9%
				10MV	NR	2.1%
				15MV	NR	3.4%
			NRCP risk co-efficients	18MV	1.7%	5.1%
			Based on maximum MU to generate “conservative maximum risk estimate”			
Schneider 2006 [41]	In field and out of field	Scanned volume: calculated from TPS (Eclipse 7.3.10)	Organ equivalent dose	*Risk shown is increased risk relative to 15MV 3D-CRT*		
			LE	6MV	15%(LE) 1%(LP)	
		Photon scatter and neutrons: measured in phantoms- previously published data	LP	15MV	20% (LE) 2% (LP)	
				18MV	60% (LE) 30% (LP)	
Kry 2007 [50]	Out of field	Measured in phantom- previously published data	Organ specific dose equivalents for photons and neutrons	*(Risk ratio relative to 18MV 3D-CRT and 90% CI in parentheses)*		
			EPA risk co-efficients	6MV	with 1.9% (RR:1.4;1.1-1.8)	
			Based on average MU	10MV	1.5% (RR: 1.1; 0.9-1.3)	
				15MV	2.2% (RR: 1.6; 1.3-2.0)	
				18MV	3.6% (RR: 2.7; 2.1-3.2)	
Schneider 2007 [49]	In field and out of field	Scanned volume: calculated from TPS (Eclipse 7.3.10)	Organ equivalent dose	*Increased risk for 100Gy IMRT relative to 70Gy 15MV 3D-CRT*		
			LNT	6MV	18.4% (LE), 15.0% (LP), 22.3% (LNT)	
			LE	15MV	25.3% (LE), 17.0% (LP), 14.1% (LNT)	
		Photon scatter and neutrons: measured in phantoms- previously published data	LP			
Stathakis 2007 [45]	Primary and out of field photons	Calculated using Monte Carlo simulations (EGS4/MCSIM) using whole body CT scans	Whole body dose equivalent	6MV	2.40% (LNT)^**^	3.55% (LNT)
			NCRP risk coefficients		1.13%(LP)	2.65%(LP)
					0.93%*	2.46%*
	Neutron contribution not included-likely to increase risk by 4-10%		LNT	10MV	3.43% (LNT)	4.19%(LNT)
			LP		1.72%(LP)	3.19% (LP)
			Out-of-field component only		1.54%*	3.02%*
				18MV	3.54% (LNT)	4.05% (LNT)
					1.73%(LP)	3.07% (LP)
					1.56%*	2.90% *
Ruben 2008 [42]	In field and out of field	Scanned volume: calculated from TPS Plato RTS v1.8 and Plato-ITP v2.5)	DVH analysis	18MV (3D-CRT)	1% (2.1%) (LP)	
			Data in parentheses shown for no correction for PCa patients with long term survival (i.e. no reduced weighting)		0.8% (1.5%)(LE)	
		Rest of body: measured in phantom Neutrons: measured in phantoms- previously published data				
			LP	6MV (IMRT)		0.8% (1.7%) (LP)
			LE			
						0.6% (1.1%) (LE)
Stathakis 2009 [46]	In field and out of field	Calculated using Monte Carlo simulations (EGS4/MCSIM) using whole body CT scan	Whole body effective dose equivalent.	6MV	2.61%^**^	3.39%
				10MV	2.48%	3.09%
	Neutron contribution not included		NRCP risk co-efficients	18MV	2.24%	2.84%
			LNT			
Bednarz 2010 [33]	Out of field	Calculated using Monte Carlo simulations (MCNPX) using computational phantom	BEIR VII co-efficients	18MV (3D-CRT: 4 field box + 6 field boost, anterior-posterior 4 field of box delivered using 6MV, rest of fields 18MV)	Risk of second tumour in:	
					Stomach: 0.03%	
					Colon: 0.3%	
					Oesophagus: 0.07%	
					Thyroid: 3.1X10^-4^%	
				6MV IMRT		Risk of second tumour in:
						Stomach: 0.04%
						Colon: 0.4%
						Oesophagus: 0.07%
						Thyroid: 1.92X10^-4^%
Patil 2010 [62]	In field	Scanned volume: calculated from TPS (Eclipse 7.3.10)	Organ equivalent dose	6MV	No comparator	Modal estimate per 10000 person years:
			LP			
						Bladder: 0.1
						Rectum: 3.42
						Small intestine: 7.789
						(Whole body: 129.95***)

¥Patients were treated with pelvic fields for rectal and gynaecological cancers. As prostate cancer patients may also be treated with pelvic RT, this study has been included.

§Figures shown for wedged conventional fields.

*estimations excluding dose to rectum and bladder, i.e. out of field component only.

** conventional four field box.

***: mixed population; includes patients treated for prostate cancer (n = 8) and head and neck cancer (n = 10).

*LP*: linear plateau *LE*: linear exponential *LNT*: linear no threshold; *DVH*: dose volume histogram.

*TPS*; treatment planning system; *BEIR*: Biologic Effects of Ionizing Radiations.

*NR*: not reported. *RR*: risk ratio. *CI*: confidence interval.

Studies comparing RISPC risk at equivalent energies have consistently shown an increase in risk with IMRT. This has largely been attributed to the increase in leakage as a result of increased MU requirements [2,45]. In addition, the increased volume of normal tissue irradiated to a low dose may contribute, although as mentioned above, this is a matter of debate [2,37,42]. Instead of comparing similar energies, the studies by Bednarz et al and Ruben et al, compared higher energy (18MV) conformal treatments with lower energy (6MV) IMRT treatment, and found risks to be comparable [33,42]. Their comparisons are valid, as in practice conformal plans will often employ higher energies while IMRT is often delivered using 6MV. It is recognised that at higher energies there is an increased contribution to out-of-field radiation from neutron production. The size of this contribution and thus the absolute impact RISPC risk, is a matter of debate as a result of uncertainties regarding the radiation weighting factor which should be applied to neutrons and differences in the depths at which neutron doses are measured [45-51]. The potential increase in RISPC risk from higher energy photons may partly explain the lack of difference in risk observed by Bednarz et al and Ruben et al. Comparing estimated RISPC risk from higher energy (15 or 18MV) 3D-conformal techniques with 6MV IMRT in the other studies produces mixed results with some studies and dose–response models (LP) estimating similar levels of risk [48], some estimating reduced risks [47] and some estimating a persistently increased risk from 6MV IMRT [40,45,46,49].

Ruben et al, as above, estimated that risks were similar between 18MV 3D-CRT and 6MV IMRT based on LP and LE models [42]. The group found that despite the increased volume of tissue irradiated to low dose with IMRT, this did not result in significant increases in RISPC risk in tissues within the DVH volume. The group suggested that this could be because the smaller field sizes and less than 100% beam intensity employed when delivering IMRT result in reduced scatter within the patient and from the machine head, which would compensate for increased leakage as a result of increased MU requirements. The group also suggested that the number of MU required to deliver IMRT, was partly dependent on the software and hardware used. As such, different hardware/software combinations could result in increased MU requirements and therefore increased leakage, which could outweigh any reduction in risk from reduced scatter secondary to smaller field sizes and reduced beam intensity [42]. The group went on to further investigate out-of-field dose from IMRT compared to 3D-CRT (albeit using tonsillar radiotherapy plans) and demonstrated that, as they had suggested, the improved conformity of IMRT resulted in an 11% reduction in within patient scatter (the effect of which predominated up to 10 cm from the field edge) but a five times increase in collimator scatter as a result of increased beam on time (the effect of which predominated over 10 to 20 cm from the field edge) and a three times increase in head leakage due to increased MU requirements (which predominated beyond 20 cm from the field edge) [52]. Overall, therefore, IMRT resulted in a 1.8 times increase in out-of-field dose but, importantly, in absolute terms, this increase in dose was very small and equivalent to only 0.14% of the prescription dose [52]. Again, the group suggested that the proportional and absolute differences in out-of-field dose were dependent on the hardware and software combinations used, as well as the field sizes employed [52].

### Impact of protons vs. photon IMRT or 3D-CRT

Studies estimating RISPC risk following proton treatments have consistently shown a reduction in risk compared with 3D-CRT and IMRT, regardless of whether spot or passive scanning techniques are used (Table 2). The reduction in risk can be considerable: Yoon et al estimated out-of-field RISPC risk from protons to be about one fifth of that with IMRT, and the risk of rectal or bladder cancers to be approximately halved [53]. The reduction in risk was largely the result of reduced dose to non-target tissues as a result of the high conformity of proton treatments which results from reduced exit doses, which result in a reduction in the volume of normal tissue irradiated [36]. Close to the field, there was a reduction in secondary radiation with proton compared to photon treatments, while at increased distances, the secondary doses from protons were higher, largely due to neutron production within the patient and machine head. The only exception to the above, was demonstrated by Fontenot et al when a weighting factor of 5 was applied to the neutron absorbed dose [28]. With this weighting, RISPC risk becomes comparable between proton and photon treatments. A weighting factor of 5, however, is not supported by most current evidence [31,54], and so lower weighting factors, all of which resulted in reduced risk estimation with protons, can be considered more realistic. Fontenot et al also calculated uncertainties associated with risk estimates. In terms of ratios of excess relative risk (another modelling process which incorporates the effects of fractionation [55-57]), there were only small uncertainties related to the dose–response model employed, while neutron weighting and inter-patient variability resulted in larger uncertainties [28]. Overall, uncertainties were in the order of +/−33%.

**Table 2:** Risk of second fatal malignancy using protons compared to photon treatments

**Study**	**Region assessed**	**Method of obtaining dose data**	**Method of risk calculation**	**Type of protons (SS or PS)**	**Risk of second malignancy from protons and comparator**
Schneider 2006 [48]	Primary and out-of-field	Proton dose :calculated by TPS (PSI proton treatment planning program)	Organ equivalent doses	SS	Approximately 50% reduction in risk of second cancer with SS protons compared to 15MV 3D-CRT using both LE and LP models
			LE		
		Neutron dose: measured in phantom- previously published data	LP		
Schneider 2007 [49]	Primary and out-of field	Proton dose :calculated by TPS (PSI proton treatment planning program)	Organ equivalent dose	SS	Risk from 100Gy protons relative to 70Gy 15MV 3D-CRT:
			LNT		**−**40.7% (LE)
		Neutron dose: measured in phantom- previously published data	LE		**−**41.3% (LP)
			LP		**−**40.0% (LNT)
Fontenot 2009 [36]	Primary and out-of-field	Primary dose: calculated by TPS (Eclipse).	Equivalent doses	PS	Compared to 6MV step and shoot IMRT, ratio of excess relative risk with protons:
			Ratio of excess relative risk (RRR)		0.61 (small patient)
		Proton scatter: Monte Carlo simulations- previously published	BEIR organ specific risk co-efficients		0.66 (medium patient)
					0.74 (large patient)
		Photon scatter:			
		Measured in phantom- previously published data	LNT**		
Fontenot 2010 [28]	Primary and out-of-field	Primary dose: calculated by TPS (Eclipse).	As above	PS	Compared to 6MV step and shoot IMRT, ratio of excess relative risk with protons:
			Weighting for neutrons also varied		
		Proton scatter: Monte Carlo simulations- previously published			0.66 (95%CI: 0.63-0.69; neutron weighting 1)
					0.61 (95%CI: 0.59-0.63; neutron weighting 0.5)
					0.75 (95%CI: 0.72-0.78; neutron weighting 2)
		Photon scatter: Measured in phantom- previously published data			1.03 (95%CI:0.99-1.07; neutron weighting 5)
					Total uncertainty in region of +/−33%
Yoon 2010 [53]	Out-of-field	Measured in phantom	Organ equivalent dose	PS	Relative risk compared to 6MV IMRT:
					Stomach: 0.15
			LP		Lung: 0.17
					Thyroid: 0.10
					Bladder: 0.40
					Rectum: 0.51

*SS*: spot scanned; *PS*: passive scanning.

**: LP and LE models also used but results not reported other than similar outcomes with all dose–response models; LNT: linear no threshold; LP: linear plateau; *LE*: linear exponential; *BEIR*: Biologic Effects of Ionizing Radiations; *CI*: confidence interval.

It should be noted, however, that although RISPC risk from protons was lower compared to IMRT using both spot and passive scanning techniques, passive scanning techniques result in much greater neutron production and so any reduction in RISPC risk might be less with spot than passive scanning techniques. Of the studies reviewed here, none have directly compared RISPC risk from spot and passive scanning techniques. In addition, spot scanning has only been compared to 3D-CRT, while passive scanning has only been compared to IMRT. Accepting the limitations in the comparison, however, the reductions in RISPC risk using spot scanning compared to 3D-CRT are in the region of 40 to 50%, while when comparing passive scanning to IMRT (and when employing realistic neutron weighting factors), smaller RISPC risk reductions, in the region of 25 to 40%, are observed [28,36,48,49].

### Impact of tomotherapy

Followill et al estimated out-of-field RISPC risk from tomotherapy. This study examined pelvic radiotherapy for rectal and gynaecological primary tumours, but remains relevant for PCa patients receiving pelvic treatments [47]. Risk appeared larger than those estimated from conventional RT or IMRT at equivalent photon energies. At 6MV the estimated risk was 2.8%, and increased to 13.1% and 24.4% at 18MV and 25MV respectively. Only out-of-field radiation was considered, and if the primary beam contribution was also included, risks might lessen [37].

### Impact of BT

RISPC risk following BT was estimated by Takam et al [58]. The group employed the competitive risk model to estimate risks from differential DVHs for the rectum and urethra. Estimates were calculated for LDR monotherapy (I-125), HDR monotherapy (Ir-192), and combination 3D-CRT with HDR boost (Ir-192). Considering the rectum and urethra, with LDR-BT, estimated risks were 2.0x10^-4^% ± 3x10^-4^ and 1.3x10^-8^% ± 7x10^-8^respectively, and for HDR monotherapy were 1.0x10^-4^% ±1x10^-4^ and 2.3x10^-8^% ±7x10^-8^. For EBRT-BT, rectal cancer risk was estimated at 0.06%. Overall, the lowest RISPC risks were associated with HDR or LDR BT monotherapy, and were attributed to the high (cell sterilising) equivalent doses received by small regions of neighbouring organs [58]. Unfortunately the group were unable to also estimate the risk of bladder RISPC as the ultrasound planning system did not include the whole bladder volume [58]. In addition, it should be noted that this study examined RISPC risk for the rectum and urethra only, and not for all organs or the whole body, as has been done in other studies, and so direct comparisons with other studies which have estimated whole body or all organ risk should be performed with caution.

### Impact of arc treatments

Alvarez Moret et al examined RISPC risk from quasiIMAT (intensity modulated arc therapy), a pseudo-rotational techniques employing 36 equally spaced step and shoot beams to simulate an arc [59]. Estimates were calculated for quasiIMAT and IMRT using 36 and 72 segments. OED (which is proportional to RISPC risk) was used, employing LP and LE models, to compare techniques. OED was similar using both models. For both IMRT and quasiIMAT, a higher number of segments resulted in higher OED outside the scanned area (i.e. out-of-field). Most OED came from the primary beam (88% with IMRT, 86% with quasiIMAT). OED was similar with 36 segment quasiIMAT and IMRT. When 72 segments were used there was a small increase in OED with quasiIMAT but this was not considered significant. The increase was the result of increased MU requirements (causing increased leakage) to deliver 72 segment quasiIMAT. Despite the increase in volume of normal tissue irradiated to a low dose due to the large number of beams with quasiIMAT, overall, quasiIMAT did not significantly increase RISPC risk [59].

More recently, albeit out with the time-frame of our search, but included as the only study to examine second cancer risks associated with actual arc treatements, is the work by Rechner et al [60]. This group compared the risks of bladder and rectal RISPC from proton arc therapy and photon volumetric modulated arc therapy (VMAT) by calculating ratios of excess relative risks. DVH data was used to provide details of the therapeutic dose and out-of-field information was obtained using previously published data for VMAT, and Monte Carlo simulations for proton arc therapy. The LNT, LE and LP models were employed and two different inflexion points (i.e. the dose beyond which risk is no longer linear with dose) were examined for the LE and LP models. Proton arc therapy was found to predict significantly lower risks of second bladder or rectal cancer according to LE and LP models with the ratio of excess relative risk (proton arc therapy:VMAT) as 0.74 and 0.86 using the LE model with inflexion points after 10Sv and 40Sv respectively, and 0.84 and 0.91 using the LP model with inflexion points after 10Sv and 40Sv respectively [60]. There was no significant difference in second rectal or bladder cancer risk when using the LNT model. The group also compared the calculated excess relative risk of second bladder and rectal cancer from in-field radiation using proton arc therapy and VMAT with that previously estimated for IMRT and lateral opposed protons by Fontenot et al [36]. Numerically, and using a LNT model, VMAT resulted in lower risks of second bladder and rectal cancer compared to IMRT (excess relative risk for bladder RISPC: 5.25 with VMAT and 8.88 with IMRT, excess relative risk for rectal RISPC: 2.09 with VMAT and 3.32 for IMRT). Proton arc therapy resulted in slightly higher risks of second bladder or rectal cancer compared to lateral-opposed proton therapy (excess relative risk for bladder: 4.86 with proton arc and 3.68 with lateral-opposed protons, excess relative risk for rectum: 2.74 with proton arc and 2.01 for lateral-opposed protons) [60].

### The impact of the primary and out-of-field doses

As above, studies considering out-of-field doses alone, and thus neglecting the impact of the primary beam, have been criticised [37,41]. Omitting the primary beam contribution (and accompanying cell sterilising doses) potentially results in over-estimation of RISPC risk if it is the case that at higher doses the risk of RISPC plateaus or decreases. Equally, however, if RISPC risk were to continue to increase beyond 4 Gy or so, then omitting the impact of the primary radiation dose would result in a falsely low RISPC risk being calculated. Thus the risk model adopted influences the effect of omitting the primary radiation beam. Schneider et al, using OED, demonstrated that omitting the effect of the primary beam resulted in an over-estimation of risk by a factor of about 2 when considering a 15MV IMRT plan relative to a conventional 18MV plan [41]. In contrast, Kry et al, calculated RISPC risk for 6MV and 18MV IMRT plans also using OED, and demonstrated that risk remained similar to that estimated using out-of-field dose alone [39]. The reasons for these differences were not fully explained.

### Other factors

Other factors which have been examined with regard to RISPC risk include dose escalation, hypofractionation, CTV-PTV margin width, collimator angles and photon energies [40,45-51,58,61]. Detailed discussion is beyond the scope of this article.

## Discussion

All studies acknowledge that there are uncertainties and limitations in estimating RISPC risk. As such, absolute values for risk are perhaps less useful than comparisons between values obtained using the same method. Dose–response models which encompass the effects of the primary dose as well as out-of-field doses are considered more realistic than models only dealing with out-of-field risks which may over-estimate or under-estimate RISPC risks depending on the dose–response model employed and the actual doses received by tissues [37,59]. For similar energies, several studies suggest that IMRT results in increased RISPC risk. This has often been attributed to an increase in MU requirements and head leakage. Indeed, it has been shown that, compared to 3D-CRT IMRT does result in increased leakage. Furthermore, increased beam on time results in increased collimator scatter, both of which contribute to an increase in out-of-field dose [52]. IMRT also results in a reduction in within patient scatter as a result of improved conformity and this potentially offsets some of the increase in risk as a result of increased leakage and collimator scatter [52]. Overall, however, out-of-field dose from IMRT does appear to be increased compared to 3D-CRT, but in absolute terms the increase in dose, and thus any increase in RISPC form out-of-field dose, is potentially very small [52]. The relative impact of all these factors depends on the software/hardware combinations and field sizes employed [42]. The increased volume of normal tissue receiving low doses with IMRT has also been thought to contribute to increased RISPC risk in tissues within the DVH volume but the extent to which this contributes is influenced by the dose–response model employed and may, in fact, also be very small [41,42,59]. While 3D-conformal treatments often use higher energies to increase penetration (and thus result in neutron production, contributing to RISPC risk), IMRT generally uses 6MV, and so the comparison between 3D-CRT and IMRT should not be restricted to equivalent energies alone.

Despite uncertainties, in general, the absolute risk of RISPC from IMRT appears small, particularly when estimated with dose models encompassing primary and out-of-field doses [42,48,62]. Although follow-up and patient numbers are limited, clinical data supports this suggestion: Huang et al, within a matched pair analysis, demonstrated that patients treated with IMRT or 3D-CRT compared to surgically treated patients were not at increased risk of second primary cancers, while patients treated with 2D radiotherapy were at increased risk of second cancers overall as well as bladder and lymphoproliferative cancers specifically [13].

Studies involving proton treatments have consistently shown reduced RISPC risks compared to 3D-CRT and IMRT, largely because a reduction in exit doses results in a reduction in the volume of normal tissues irradiated, thus resulting in improved conformity [28,36,48,49] Similarly, the risk of RSIPC has been shown to be lower with proton arc therapy compared to photon VMAT [60]. Brachytherapy is associated with very low estimated risks of second rectal cancers [58]. Compared to IMRT, limited evidence suggests tomotherapy is associated with higher estimated RISPC risks, while photon arc therapies (simulated or otherwise) are not [47,59,60].

The ALLEGRO project (full title: Early and late health risks to normal/healthy tissues from the use of existing and emerging techniques for radiation therapy) is a collaborative project involving 13 European organisations which aims to clarify some of the uncertainties surrounding modelling and measurement of radiation doses [63]. The project will ultimately produce clinical recommendations regarding the RISPC risks associated with current and emerging radiation techniques.

## Conclusions

In summary, multiple factors are involved when estimating RISPC risk, and there are uncertainties in all estimations. At present follow-up from clinical studies is too short, or patient numbers too small, to determine if the estimated changes in risk from more modern irradiation techniques translate into clinically significant changes in second primary cancer incidence in practice. Until then, the warnings produced from planning studies must be borne in mind when considering which patients to irradiate, and which technique to employ.

## Abbreviations

3D-CRT: Three Dimensional Conformal Radiotherapy; BEIR: Biologic Effects of Ionizing Radiations; CI: Confidence interval; DDREF: Dose And Dose Rate Effectiveness Factor; IMAT: Intensity Modulated Arc Therapy; IMRT: Intensity Modulated Radiotherapy; LE: Linear Exponential; LNT: Linear No Threshold; LP: Linear Plateau; MU: Monitor Units; MV: Megavolts; NRCP: National Council on Radiation Protection and Measurements; OED: Organ Equivalent Dose; PCa: Prostate Cancer; PA: Passive Scattering; RISPC: Radiation Induced Second Primary Cancer; RR: Risk ratio; SS: Spot Scanning; TPS: Treatment Planning System; VMAT: Volumetric Modulated Arc Therapy.

## Competing interests

The authors have no competing interests to declare.

## Authors’ contributions

AH, PH, FAS and JV conceived and designed the study. LM undertook the review. All authors participated in the drafting and revising of the manuscript.

## Supplementary Material

Additional file 1 Search Strategy. Click here for file
